# Genetic structure of Tibetan populations in Gansu revealed by forensic STR loci

**DOI:** 10.1038/srep41195

**Published:** 2017-01-23

**Authors:** Hong-Bing Yao, Chuan-Chao Wang, Jiang Wang, Xiaolan Tao, Lei Shang, Shao-Qing Wen, Qiajun Du, Qiongying Deng, Bingying Xu, Ying Huang, Hong-Dan Wang, Shujin Li, Bin Cong, Liying Ma, Li Jin, Johannes Krause, Hui Li

**Affiliations:** 1 Key Laboratory of Evidence Science of Gansu Province, Gansu Institute of Political Science and Law, Lanzhou 730070, China; 2 State Key Laboratory of Genetic Engineering and Ministry of Education Key Laboratory of Contemporary Anthropology, Collaborative Innovation Center for Genetics and Development, School of Life Sciences, Fudan University, Shanghai, 200433, China; 3 Department of Archaeogenetics and Eurasia3angle research group, Max Planck Institute for the Science of Human History, Kahlaische Straße 10, 07745 Jena, Germany; 4 College of Animal Sciences and Veterinary Medicine, Henan Agricultural University, Zhengzhou 450002, Henan Province, China; 5 Key Laboratory of Forensic Genetics, Institute of Forensic Science, Ministry of Public Security, Beijing, 100038, China; 6 Lanzhou University Second Hospital Clinical Laboratory, Lanzhou 730000, Gansu Province, China; 7 Department of Anatomy, Guangxi Medical University, Nanning 530021, China; 8 School of Forensic Medicine, Kunming Medical University, Kunming, 650500, China; 9 Medical Genetic Institute of Henan Province, Henan Provincial People’s Hospital, People’s Hospital of Zhengzhou University, Zhengzhou, China; 10 Hebei Key Laboratory of Forensic Medicine, Department of Forensic Medicine, Hebei Medical University, Shijiazhuang, 050017, China; 11 CAS-MPG Partner Institute for Computational Biology, Shanghai Institutes for Biological Sciences, Chinese Academy of Sciences, 200031 Shanghai, China

## Abstract

The origin and diversification of Sino-Tibetan speaking populations have been long-standing hot debates. However, the limited genetic information of Tibetan populations keeps this topic far from clear. In the present study, we genotyped 15 forensic autosomal short tandem repeats (STRs) from 803 unrelated Tibetan individuals from Gansu Province (635 from Gannan and 168 from Tianzhu) in northwest China. We combined these data with published dataset to infer a detailed population affinities and genetic substructure of Sino-Tibetan populations. Our results revealed Tibetan populations in Gannan and Tianzhu are genetically very similar with Tibetans from other regions. The Tibetans in Tianzhu have received more genetic influence from surrounding lowland populations. The genetic structure of Sino-Tibetan populations was strongly correlated with linguistic affiliations. Although the among-population variances are relatively small, the genetic components for Tibetan, Lolo-Burmese, and Han Chinese were quite distinctive, especially for the Deng, Nu, and Derung of Lolo-Burmese. Han Chinese but not Tibetans are suggested to share substantial genetic component with southern natives, such as Tai-Kadai and Hmong-Mien speaking populations, and with other lowland East Asian populations, which implies there might be extensive gene flow between those lowland groups and Han Chinese after Han Chinese were separated from Tibetans. The dataset generated in present study is also valuable for forensic identification and paternity tests in China.

The Sino-Tibetan languages, spoken by over a billion people all over East Asia and Southeast Asia, have been classified into two subfamilies, namely Chinese and Tibeto-Burman^1^. The linguistic connection between Chinese and Tibeto-Burman are well established. Chinese was suggested to split away from Tibeto-Burman around 6 thousand years ago (kya) based on lexical evidence^2^.

During the past two decades, genetic evidence, especially from the maternal mitochondrial DNA (mtDNA) and the paternal Y chromosome, has shed more light on the history of Sino-Tibetan populations. MtDNA evidence reveals a northern Asian origin of Tibetans, due to the high frequencies of northern Asian specific haplogroup A, D, G, and M8^3^,^4^,^5^. The genetic relics of the Late Paleolithic ancestors of Tibeto-Burman populations have also been reported, such as haplogroup M62^5^. Y chromosome suggested Tibeto-Burman populations are an admixture of the northward migrations of East Asian initial settlers with haplogroup D-M175 in the Late Paleolithic age, and the southward Di-Qiang people with dominant haplogroup O3a2c1*-M134 and O3a2c1a-M117 in the Neolithic Age^6^,^7^,^8^. Haplogroup O3a2c1*-M134 and O3a2c1a-M117 are also characteristic lineages of Han Chinese, comprising 11.4% and 16.3%, respectively^9^,^10^. However, another dominant paternal lineage of Han Chinese, haplogroup O3a1c-002611, is found at very low frequencies in Tibeto-Burman populations, suggesting this lineage might not have participated in the formation of Tibeto-Burman populations^6^,^9^,^10^,^11^. Sex-biased admixture has also been observed during the formation of Tibeto-Burman populations. Southern Tibeto-Burman populations exhibit a stronger influence of northern immigrants on the paternal lineages and a more extensive contribution of southern natives to the maternal lineages^12^. Likewise, the southern natives have made a greater contribution to the maternal lineages of southern Han Chinese^13^. Tibeto-Burman populations tend to cluster with North Asian and Tai-Kadai populations rather than Han Chinese based on the frequency data of 15 autosomal short tandem repeats (STRs)^14^. A genome-wide study of PanAsia SNP project reveals that Han Chinese populations show varying degrees of admixture between a northern Altaic cluster and a Sino-Tibetan/Tai-Kadai cluster^15^. But Tibetan populations were not included in the PanAsia project. The analyses of more than 30 deeply sequenced genomes of Tibetans in Tibet Autonomous Region give consistent results with Y chromosomes that most of the Tibetan gene pool diverged from that of Han Chinese about 15 kya to 9 kya. The shared ancestry of Tibetan-enriched sequences dates back to 62–38 kya, representing Paleolithic colonization of the plateau^16^. An ancient DNA-based study using ancient Nepalese genomes of the Chokhopani, Mebrak and Samdzong sites spanning 3 to 1 kya demonstrates that the Tibetan Plateau experienced millennia of genetic continuity which continues until the present day^17^.

From previous studies, the origin of Sino-Tibetan populations seems to involve substantial genetic admixture with surrounding populations. However, the limited markers of mtDNA and Y chromosome and small sample sizes and insufficient sampling of genome-wide study are far from enough to give a comprehensive understanding about the genetic history and admixture process of Sino-Tibetan populations. In addition, Tibetan populations of Gansu province, the key area for the diversification of Amdo Tibetans, have seldom been studied genetically. Therefore, we analyze 15 autosomal STRs in 635 and 168 unrelated individuals from two Tibetan populations in Gannan and Tianzhu of Gansu province to explore the genetic structure of Tibetan populations in northwest China and to test population affinities and the level of admixture of Sino-Tibetan populations with surrounding populations.

## Methods

We collected blood samples of 635 and 168 unrelated individuals from two Tibetan populations in Gannan and Tianzhu, Gansu province. Our study was approved by the Ethnic Committee of Gansu Institute of Political Science and Law. The study was conducted in accordance with the human and ethical research principles of Gansu Institute of Political Science and Law. All individuals were adequately informed and signed their informed content before their participation. For each sample, genomic DNA was extracted according to the Chelex-100 method and proteinase K protocol^18^. The 15 most widely used forensic loci were amplified simultaneously using AmpFlSTR Sinofiler PCR Amplification Kit (Applied Biosystems, Foster City, CA, USA) at the D8S1179, D21S11, D7S820, CSF1PO, D3S1358, D13S317, D16S539, D2S1338, D19S433, vWA, D18S51, D5S818, FGA, D6S1043 and D12S391 STR loci. The PCR products were analyzed with the 3500XL DNA Genetic Analyzer and Genemapper ID-X software (Applied Biosystems, Foster City, CA, USA).

Allele frequency, heterozygosity, polymorphism information content (PIC), discrimination power (DP), probability of paternity exclusion (PPE) were calculated using PowerStatesV12 (http://www.promega.com/). Tests for Hardy–Weinberg equilibrium were performed in Arlequin v3.5.1.3^19^. Since the statistical analyses in this study were on the basis of Bayesian-clustering algorithm, raw genotypic data of 13 STRs (excluding D6S1043 and D12S391) from 59 populations all around the world were extracted to determine population affinity^14^,^20^,^21^,^22^,^23^,^24^,^25^,^26^,^27^,^28^,^29^,^30^,^31^,^32^,^33^,^34^,^35^,^36^,^37^,^38^,^39^,^40^,^41^,^42^,^43^,^44^,^45^,^46^,^47^,^48^,^49^,^50^. Analysis of molecular variance (AMOVA), average number of pairwise differences, pairwise Fst, Slatkins linearized Fst, and coancestry coefficients were all calculated in Arlequin v3.5.1.3^19^ using genotype data. The detailed population genetic structure was performed using model-based clustering method implemented in Structure 2.3.4^51^,^52^ under assumptions of admixture, LOCPRIOR model, and correlated allele frequencies. Each run used 100,000 estimation iterations for K = 2 to 12 after a 20,000 burn-in length with several replicates. Posterior probabilities for each K were computed for each set of runs. Graphical display for Matrix plot of genetic distance and population structure were carried out in R statistical software v3.0.2^53^ and Distruct v1.1^54^.

## Results

### Forensic parameter analysis

Fifteen STR loci were genotyped in two populations sampled from Gannan and Tianzhu of Gansu province and their allele frequencies along with a number of genetic and forensic parameters of interest are provided in Supplementary Table 1 and 2. No significant deviation was observed for Hardy–Weinberg equilibrium tests, indicating that our samples well represent the populations. The loci in both populations were highly discriminating with DP ranging from 0.852 to 0.974, demonstrating that those loci are useful for forensic identification.

### Interpopulation genetic distances

**Figure 1:**
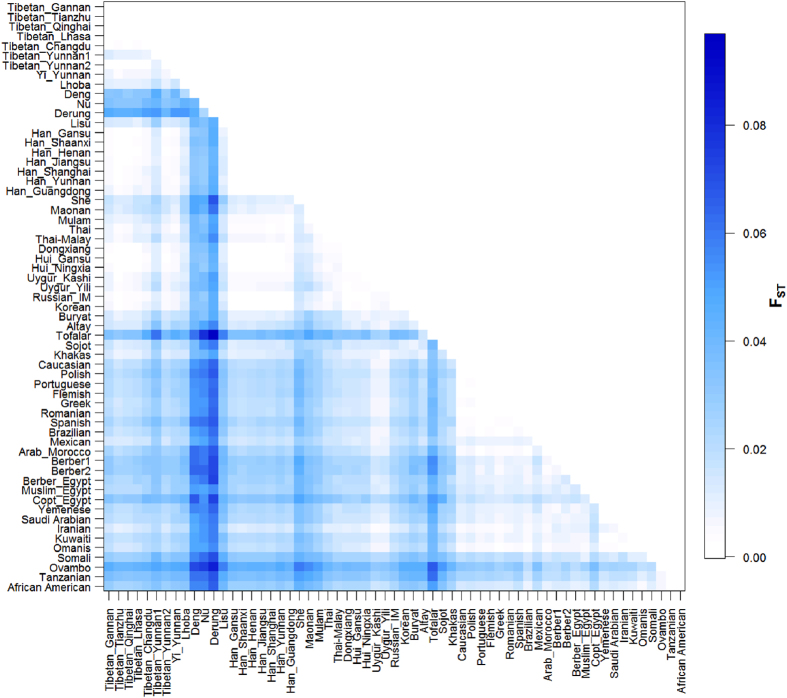
Plots of pairwise Fst of Tibetan in Gannan, Tianzhu and other 59 worldwide populations.

We performed various parameters of genetic diversity and distances to infer population structure between Tibetans in Gannan and Tianzhu, as well as compared them with previously studied populations. The Tibetans in Gannan and Tianzhu fall into the general profile of Tibetan groups, showing extremely small genetic distances with other Tibetan populations. The within-population component of genetic variation, estimated here as 99.14% (Table 1), accounts for most of genetic diversity of the 20 Sino-Tibetan populations. The small among-population and among-group variance components support the genetic affinity among the Sino-Tibetan populations. The pairwise Fst comparisons in Fig. 1 also confirm the genetic similarity between Tibetan and Han Chinese populations, with almost all the values below 0.01. However, Deng, Nu, and Derung seem like to be outliers of the Sino-Tibetan profile due to almost all the Fst values between each of them with other Sino-Tibetan populations are above 0.03. The Fst between other Lolo-Burmese populations with Tibetan or Han Chinese were also slightly higher than the values between Tibetan and Han Chinese. Tibetan and Han Chinese also show close genetic relationship with Russian samples collected from Inner Mongolia in north China and Korean in East Asia, but not with the population in South Siberia, such as Buryat, Altay, Tofalar, Sojot, and Khakas. The genetic distances between Tibetan and Han Chinese with Tai-Kadai speaking (Maonan, Mulam, and Thai) or Hmong-Mien speaking (She) populations were also larger than those between Tibetan and Han Chinese. The Muslim populations in northwest China exhibited relatively small genetic distances with Tibetan and Han Chinese, revealing the substantial gene flow from Sino-Tibetan populations into Muslim people during their Islamization. Average number of pairwise differences, Slatkins linearized Fst, and coancestry coefficients also reveal a very similar pattern with the pairwise Fst (Supplementary Table 3).

### Clustering by structure analysis

**Figure 2:**
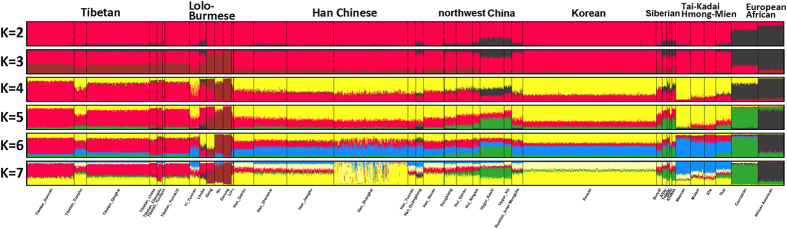
Estimated population genetic structure of Tibetan in Gannan and Tianzhu and other 36 worldwide populations. Note that the bold names “Tibetan”, “Lolo-Burmese”, “Han Chinese”, “northwest China”, “Korean”, “Siberian”, “Tai-Kadai Hmong-Mien”, “European”, and “African” refer to the group classifications of present-day populations based on language and geographic affinity. Those names are not the labels for the inferred ancestral population in Structure analysis.

We then applied a model based clustering algorithm in Structure to infer the detailed genetic ancestry at individual level. This approach will place individuals into K clusters, where K is set in advance but can be varied. The results for K = 2 to 7 are shown in Fig. 2 and Supplementary Table 4. At K = 2, a clear distinction is observed between the present-day Europeans and Africans with populations from Asia. At K = 3, a component maximized in present-day Lolo-Burmese populations separates especially Deng, Nu, and Derung from other Asian populations. At K = 4, a component maximized in present-day Tibetan populations appears, which also comprises about 20–50% of Han Chinese and southern Siberian populations, but is greatly reduced in present-day Maonan samples. At K = 5, Europeans are separated from Africans. The present-day Uygur and southern Siberian populations seem to have half of this European maximized component. The next cluster at K = 6 corresponds to present-day Tai-Kadai and Hmong-Mien speaking populations in south China with a component maximized in those southern native groups. This southern native component comprises almost half of the Han Chinese and Siberian gene pool. At K = 7, the supposed southern native genetic component in present-day Han Chinese and Siberian populations forms a new cluster. It’s quite clearly from the Structure analysis that Han Chinese, Korean and populations in northwest China share the similar membership, but the Siberian groups share more genetic component with Europeans. The origin of Tibetan populations seemed to involve extensive gene flow with Han Chinese. Southern natives, such as Tai-Kadai and Hmong-Mien speaking groups, share substantial genetic component with Han Chinese, Muslim populations in northwest China, and Yi in Yunnan, but not with Tibetan populations. The Tibetans in Gannan of Gansu province are genetically very similar with Tibetans in Tibet, Qinghai and Yunnan, but Tibetans in Tianzhu of Gansu province seem to have more lowland East Asian genetic components than other Tibetan populations have.

## Discussion

The origin and diversification of Sino-Tibetan populations have become long-standing hot topics among linguists, population geneticists, anthropologists, and archaeologists. However, the limited genetic information of Tibetan populations in northwest China has made this topic far from clear. Here, we genotyped 15 forensic autosomal STRs from 635 and 168 unrelated Tibetan individuals from Gannan and Tianzhu of Gansu province, together with published forensic dataset to infer a detailed genetic structure of Sino-Tibetan populations. The Tibetans in Gannan share a very similar genetic makeup with other Tibetan populations from Tibet, Qinghai, and Yunnan. While Tibetans in Tianzhu County seem like to share more genetic component with lowland East Asians, such as Han Chinese, Muslim and Korean populations, which is understandable as those Tibetans are surrounded by Han Chinese and Chinese Muslims. The genetic structure of studied Sino-Tibetan populations is strongly correlated with linguistic affiliations, as we can detect three distinctive genetic components for Tibetan, Lolo-Burmese, and Han Chinese although the among-population variances are relatively small. The Yi of Yunnan province, one of Lolo-Burmese speaking populations, is found out to be an admixture between Tibetan, Han Chinese, and southern natives (Tai-Kadai and Hmong-Mien speaking groups). However, other Lolo-Burmese populations, such as Deng, Nu, and Derung, form a distinctive cluster, which is probably due to long-term isolations and genetic drift as those populations are all small and living on hunting and gathering^12^.

Previous studies, especially using mtDNA and Y chromosome, had suggested the North Asian origin of Tibetan populations^55^,^56^. Our results show that the Tibetans are quite distinctive from Siberian populations. The Siberian populations, such as Buryat, Altay, Tofalar, Sojot, and Khakas, share substantial genetic components with European groups which are rarely seen in Tibetan populations. The results are consistent with genome-wide evidence that there is no significant gene flow from West Eurasians into Tibetans^14^,^15^. We suspect that the proposed northern ancestral group that leaded to present-day Tibetan populations was probably separated with the lineage that later became the East Asian part of the Siberian groups earlier before the Siberian groups were extensively admixed with West Eurasian lineages. We caution that the geographical distribution of past populations is probably not accurately reflected in present-day distributions. An important direction for future work is to work out the exact phylogenetic relationship of the proposed ancient population branch leading to present-day Tibetan populations to other extant Eurasian groups by sequencing ancient samples from Tibetan Plateau and the Upper-Middle Yellow River Basin.

The genetic makeups of the Tai-Kadai and Hmong-Mien speaking populations in south China are similar with Han Chinese rather than with Tibetan groups. The Muslim populations in northwest China and Korean people also exhibit the similar component cluster pattern. The possible scenario for this observation is that those lowland populations might have extensive gene exchange with Han Chinese after Han Chinese were separated from Tibetans. The autosomal STR results are consistent with uniparental Y chromosomal and mtDNA evidence that southern natives made a greater contribution to the maternal lineages of southern Han Chinese^11^.

## Additional Information

**How to cite this article:** Yao, H.-B. *et al*. Genetic structure of Tibetan populations in Gansu revealed by forensic STR loci. *Sci. Rep.* **7**, 41195; doi: 10.1038/srep41195 (2017).

**Publisher's note:** Springer Nature remains neutral with regard to jurisdictional claims in published maps and institutional affiliations.

## Supplementary Material

Supplementary Legends

Supplementary Table 1

Supplementary Table 2

Supplementary Table 3

Supplementary Table 4

## Figures and Tables

**Table 1:** AMOVA results for 13 autosomal STRs at population and group scales.

Source of variation	d.f.	Sum of squares	Variance components	Percentage of variation
Among group	2	175.190	0.01925	0.37
Among populations within groups	17	306.945	0.02549	0.49
Within populations	11204	58131.8	5.18848	99.14
Total	11223	58613.9	5.23323	

20 Sino-Tibetan populations have been classified into 3 groups: Han Chinese, Tibetan, and Lolo-Burmese.
