# Supplementary material for: Genetic structure of Tibetan populations in Gansu revealed by forensic STR loci
Source: Sci Rep. 2017 Jan 23;7:41195. doi: 10.1038/srep41195 (PMC5255561; doi:10.1038/srep41195)
Supplement: Supplementary Legends [file srep41195-s1.doc]

**Article for Scientific Reports**

**Genetic structure of Tibetan populations in Gansu revealed by forensic STR loci**

Hong-Bing Yao^1,✝^, Chuan-Chao Wang^2, 3, ✝^, *, Jiang Wang^4^, Xiaolan Tao^1^, Lei Shang^5^, Shao-Qing Wen^2^, Qiajun Du^6^, Qiongying Deng^7^, Bingying Xu^8^, Ying Huang^8^, Hong-Dan Wang^9^, Shujin Li^10^, Bin Cong^10^, Liying Ma^1^, Li Jin^2, 11^, Johannes Krause^3^, Hui Li^2^,*

1. Key Laboratory of Evidence Science of Gansu Province, Gansu Institute of Political Science and Law, Lanzhou 730070, China
2. State Key Laboratory of Genetic Engineering and Ministry of Education Key Laboratory of Contemporary Anthropology, Collaborative Innovation Center for Genetics and Development, School of Life Sciences, Fudan University, Shanghai, 200433, China
3. Department of Archaeogenetics and Eurasia3angle research group, Max Planck Institute for the Science of Human History, Kahlaische Straße 10, 07745 Jena, Germany
4. College of Animal Sciences and Veterinary Medicine, Henan Agricultural University, Zhengzhou 450002, Henan Province, China
5. Key Laboratory of Forensic Genetics, Institute of Forensic Science, Ministry of Public Security, Beijing, 100038, China.
6. Lanzhou University Second Hospital Clinical Laboratory, Lanzhou 730000,Gansu Province, China
7. Department of Anatomy, Guangxi Medical University, Nanning 530021, China
8. School of Forensic Medicine, Kunming Medical University, Kunming, 650500, China
9. Medical Genetic Institute of Henan Province, Henan Provincial People's Hospital, People's Hospital of Zhengzhou University, Zhengzhou, China
10. Hebei Key Laboratory of Forensic Medicine, Department of Forensic Medicine, Hebei Medical University, Shijiazhuang, 050017, China
11. CAS-MPG Partner Institute for Computational Biology, Shanghai Institutes for Biological Sciences, Chinese Academy of Sciences, 200031 Shanghai, China

✝These authors contributed equally to this work.

* Corresponding author: Hong-Bing Yao, Chuan-Chao Wang and Hui Li. Tel: +86-931-7601409, E-mail addresses: yaohongb@126.com (Hong-Bing Yao); Tel: +49 (0) 3641 686-648, E-mail: wang@shh.mpg.de (Chuan-Chao Wang); Tel: +86-21-51630427, E-mail addresses: LHCA@Fudan.edu.cn (Hui Li).

**Supplementary Material**

Supplementary Table 1. 15 autosomal STRs of Tibetan in Gannan and Tianzhu.

Supplementary Table 2. Allele frequency distributions and forensic parameters

Supplementary Table 3. Matrix of pairwise F_ST_, Slatkins linearized F_ST_, average number of pairwise differences, and coancestry coefficients.

Supplementary Table 4. Estimates of posterior probabilities of data under admixture model for 38 populations and the proportion of membership of each pre-defined population in each cluster.
